# Supplementary material for: Simulation and prediction of rural population changes using agent-based modeling
Source: PLoS One. 2025 Jun 23;20(6):e0324563. doi: 10.1371/journal.pone.0324563 (PMC12184946; doi:10.1371/journal.pone.0324563)
Supplement: Attachment — Submitted filename: Response_to_Reviewers-0310.docx [file pone.0324563.s003.docx]

We sincerely thank the reviewers for their constructive comments and valuable suggestions. Our responses to each point are provided below：

**Editorial comments：**

1.There are some jargon and uncommon terms that make it difficult for a general reader to understand. Please revise these terms. For example, in line 34, "dwindling" can be replaced with "declining" for better clarity. Many technical terms are used without clear definitions (e.g., "agent rules" are vaguely described). I also noticed frequent awkward sentence structures, and some sentences are unnecessarily complex. I suggest using active voice to improve readability.

Thank you for your thorough review and valuable feedback. Concerning the issues of terminology usage and sentence structure that impact readability, we have conducted a comprehensive review of the technical terms and refined the sentence expressions throughout the manuscript. Additionally, we have sought professional guidance to ensure clarity and precision. We believe this revised version offers a more intuitive and accessible presentation.

2. Research methodology should be clearly defined in a separate section, including detailed information on data collection, methods, and analytical techniques.Table 1 contains sources, but it does not add much value to the study and can be removed or revised.

(1) This study's research methodology, which includes the agent-based simulation approach, the constructed agent behavior rules, and population validation methods, has been presented in a separate section.

(2) Table 1 presents the behavioral rules and probabilistic parameters for population agents across various age groups, derived from empirical data on birth rates, mortality rates, and migration rates obtained through rural surveys. These elements serve as a critical foundation for simulating individual agent behaviors. To ensure scientific rigor and transparency in the simulation process, we have retained this table while enhancing its content for better clarity and readability.

3.The choice of Agent-Based Modeling (ABM) is not justified. The introduction fails to critically compare ABM with other micro-level modeling approaches.There is also no discussion on why ABM is superior to alternative methods.

Concerning the insufficient rationale for choosing agent-based modeling (ABM), we have added a literature review in the introduction comparing ABM with other static simulation methods and providing additional justification for selecting this approach.

4.The study uses the Malthusian model and the Leslie model, both of which are outdated for modern demographic projections. No sensitivity analysis has been included, making the findings less reliable. The study should also include confidence intervals or robustness checks for RMSE calculations.

(1) The Malthusian model, which only predicts total population size, is somewhat outdated. In the revised version, we have removed it from the validation process. The Leslie model, a classic demographic model capable of predicting both population size and structure, is primarily used in this study to compare ABM simulation results across different age groups, thereby enhancing the cross-validation of simulation results.

(2) The key factors in this study’s simulation are birth, mortality, and migration rates. While the results section does not include a formal sensitivity analysis, the discussion section already explores simulations under high/low birth rates and high/low migration rates, effectively reflecting aspects of sensitivity analysis. In the revised manuscript, we have renamed Section 4.3 from “Discussion on Rural Population Change” to “Sensitivity Analysis and Policy Recommendations” to highlight this aspect better.

(3) **Confidence Interval:** Traditionally, RMSE is a point estimate that directly reflects the average prediction error of the entire dataset and is not typically associated with a confidence interval. In the revised version, the paired-sample T-test was used to verify the difference of the simulated and actual values of the two-village population using SPSS 27. Furthermore, Bootstrap for Paired Sampled Test was checked. The results sufficiently demonstrate that the simulated values closely match the actual values (difference equaling zero at 95% confidence level). For details, see Tables 3 and 4**.**

5.The findings are not generalizable as the study is based on only two village case studies. Additionally, the discussion section repeats points already mentioned in the results section, making it redundant. There is too much repetition throughout the paper, especially in the results and discussion sections.

(1) Although the study area consists of two village case studies, they represent two distinct types of villages—one with industrial advantages and another with industrial disadvantages. Our objective is to explore the changing patterns of the rural population using these two opposing cases, which can be extended to similar villages. In the revised manuscript, the representativeness of each village has been explicitly enhanced, and the findings from these specific cases have been applied to a broader context.

(2) Regarding content redundancy, we have streamlined the results section to avoid duplicating the same data and findings in the discussion. Instead, we focused on interpreting the underlying reasons for these results, their connections to existing studies, and their implications for future research and practice. This revision also enhances the coherence and readability of the paper.

6.The abstract and conclusion sections are weak and need to be revised to clearly highlight the study’s contributions and future research directions.

(1) Abstract: Three key points have been clarified further: (i) the differences between agent-based modeling (ABM) and traditional demographic models, emphasizing ABM’s dynamic simulation capabilities and bottom-up approach; (ii) a clear comparative explanation of population changes between the two types of villages; and (iii) an interpretation of key parameters’ sensitivity, highlighting migration and birth rates as the main drivers of rural population change. Finally, we strengthened the policy relevance by linking the research findings directly to policy recommendations, emphasizing their practical implications.

(2) Conclusion: The discussion of results has been streamlined to avoid redundancy with numerical data already presented in tables, while summarizing the population change trends in the two types of villages. The study’s contributions are explicitly highlighted, expanding on its policy implications and practical applications. Specific policy recommendations and future research directions have been provided, reinforcing the applicability of the findings to other rural areas and assessing their generalizability. Additionally, the discussion section further emphasizes the strengths and limitations of ABM in population simulation, outlining potential avenues for future methodological improvements.

7.Most of the references are based on studies from China, which limits the paper’s global relevance.

Thank you for your suggestion regarding the scope of references. To enhance the model methodology's global applicability, we have incorporated several international studies.

**Reviewer's Comments**

Reviewer #1: In this study, the authors used an agent-based method to define the attributes and behavioral rules of individual agents within a population. This approach enhances predictive capability, which is particularly valuable for policymakers seeking to address the challenges of rural population decline. The paper, in general,is well written and contains valuable material. However, several sections, including the abstract, are lengthy and need shortening.

Thank you for your recognition. The abstract has been refined to focus on the core contributions (ABM's predictive capability and policy recommendations), with redundant background descriptions removed. Other sections, such as the literature review, have been condensed to essential content. At the same time, the conclusion and discussion have been further streamlined to ensure a concise and coherent structure throughout the paper.

Reviewer #2: Thank you for writing up this manuscript on simulation and prediction of population using agent based modelling. Author discusses the caveats of traditional population models and recommends agent based modelling which is bottom up approach and based on 3 core behaviors – birth, death and migration. Author compares the results of agent based model with traditional methods and actual population and identifies agentic model output is more closed to the actual population than the other 2 models. Author explains the impact of birth, death, migration rates on the simulated output findings and discusses their impact and recommends policy suggestions how public officials can improve the birth rate and economy in rural areas.
Please find below comments:

1) Page 13: The population statistics mentioned in line 263 and 270 are for which year?

2) Page 15: Fig 4: Line 291: Figure represents Shihan village also has wider base, particularly age 10-14, line291: fewer working age group 40, but this count is still bigger than Yunfeng and bigger than elderly group in Shihan

3) Page 20: Fig 9: A & B – This figure was not discussed in the paper and predicted population of 1061 and 1324 for Yunfeng & Shihan year 2024 is not matching in this figure

4) Page 22: “Assuming birth rate increases to 3% under a policy encouraging childbirth” – during simulation birth rate is set as 7.66%. What is the reason to mention birth rate as 3% ?

(1) On page 13, the demographic data mentioned in lines 263 and 270 were obtained from the 2024 field survey.

(2) In Figure 3 (line 291), the description has been further enhanced, and it has been verified that the figure represents the age structure of the resident population in two villages based on actual survey data.

(3) On page 20, additional descriptions have been provided for parts A and B of Figure 8, explaining the simulated population changes and demographic structure changes. The data in the figure have also been verified, with detailed modifications in Figure 8.

(4) On page 22, the initial birth rate in the simulation was 7.66‰, which was later increased to 3% under the assumption of an unrestricted pro-birth policy. This adjustment aimed to explore village population changes and validate the hypothesis that "birth rate is a key factor influencing rural demographic structure," providing a basis for subsequent policy recommendations.

Reviewer #3: 1. Strengthen the Discussion of Novelty in Comparison to Past Models
Clearly state how ABM differs from and improves upon traditional models.

2. Justify Parameter Selection More Rigorously Explain why birth rates, migration probabilities, and mortality rates were chosen.

3. Improve Conciseness and Clarity in the Results Discussion Avoid repeating numbers already presented in tables; instead, summarize trends. Improve readability with subheadings and bold key findings.

4. Expand Policy Implications and Real-World Applications. Provide specific policy recommendations, not just general suggestions. Discuss how the findings can be applied to different rural contexts beyond China.

Thank you for your review. Below are my responses to your suggestions and the revisions made to the manuscript.

(1) Regarding the discussion on enhancing the novelty compared to past models, a literature review was added to the introduction section, comparing Agent-Based Modeling (ABM) with other static simulation methods. This highlights the distinctions and improvements of ABM over traditional models and explains the rationale for choosing the ABM approach. Second, the discussion was revised to emphasize the differences between Agent-Based Models (ABM) and traditional models. The advantages of ABM in capturing individual-level heterogeneity and dynamic interactions were underscored, aspects that are often oversimplified in traditional methods.

(2) Regarding stricter justification for parameter selection: The revised manuscript provides more detailed justification for selecting parameters such as birth rate, migration probability, and mortality rate. The empirical and theoretical foundations for these parameters are explained based on existing literature and relevant statistical data. Additionally, robustness tests for the model were added and included in the "Verification of simulation results" section.

(3) Improving the conciseness and clarity of the results discussion: The results section was modified to enhance clarity and conciseness in the revised manuscript. The focus was placed on summarizing key trends and policy recommendations, with the policy recommendations section bolded to improve readability and facilitate better navigation of the results. These changes are reflected in the "Sensitivity analysis and policy implications" and "Conclusions" sections.

(4) Expanding policy implications and practical applications: The revised manuscript expands on policy implications and proposes specific, actionable policy recommendations based on the research findings. Regarding population birth, it suggests lifting restrictions on rural population fertility and increasing childbirth and childcare subsidies. To address labor migration loss, it proposes supporting agricultural industries, increasing farming subsidies, enhancing land consolidation and transfer, and boosting agricultural income to attract population return and reduce outmigration. For educational migration, it recommends retaining village primary schools, slowing down school closures, increasing care for left-behind children, and investing in rural education to reduce population outflow due to education. These details can be found in the "Sensitivity analysis and policy implications" section.
