# Supplementary material for: Simulation and prediction of rural population changes using agent-based modeling
Source: PLoS One. 2025 Jun 23;20(6):e0324563. doi: 10.1371/journal.pone.0324563 (PMC12184946; doi:10.1371/journal.pone.0324563)
Supplement: Attachment — Submitted filename: Response_to_Reviewers-0424.docx [file pone.0324563.s004.docx]

We sincerely appreciate the constructive comments and valuable suggestions provided by the reviewer and editor. Our detailed responses to each point are below.

**Additional Editor Comments:**
1. The heading Experimental area, it is not appropriate for a simulation-based study; Please change it to Study area or Case study description.

The original use of the term“Experimental area”did not accurately reflect the nature of the simulation study. It has now been revised to“Overview of the study area”in the relevant heading, as reflected in the revised manuscript (see line 186).

2. The study is based on two villages, which limits the broader applicability. Please acknowledge this limitation in the discussion and limitations section, the same comment has been not addressed in revision

We agree with the possible limitations of the simulation analysis based only on two villages. However, this study focuses on two types of villages in economically underdeveloped towns and townships, respectively reflecting the two types of villages with local industrial advantages and disadvantages, as well as their population change scenarios. Perhaps the number of selected village samples is limited, but the two types of villages still have obvious representativeness in terms of industrial differences and population changes. They can reflect the characteristics of rural population evolution under industrial differences, which also exist widely in other rural areas in China.

The sample selection of this study may not reflect various types of villages and the multi-dimensional causes influencing population changes. The section of "Limitations and prospects" was added in the latest revised manuscript. However, we mainly classified the two types of villages from the perspective of rural industrial differences and deeply explored the population evolution of the two types of villages triggered thereby. The focus of the dimension analyzed in this study lies in the impact of industrial differences on the population, which is somewhat universal in rural areas across the country.

We believe that the constructed simulation framework and method are universal in villages with obvious industrial differences and their population evolution, and can provide theoretical references and modeling paradigms for the population evolution and prediction of other villages with similar industrial economic differences.

3. Several sentences still remain verbose and repetitive; the same comment was given in the last revision, but has not been appropriately addressed. Please revise the language carefully and remove all unnecessary wording. Cut down each and make it clear and short.

We have carefully reviewed the entire manuscript, with a focus on checking and streamlining lengthy, repetitive, or unclear sentences. In particular, we have comprehensively optimized the language in the sections of theoretical foundation, model design, and analysis & discussion by addressing redundant wording. This revision removed unnecessary conjunctions, repetitive expressions, and explanatory sentences to make the sentence structure more compact and concise.

4. Figures 9 to 12 lack sufficient explanation. Please ensure that each figure is discussed in detail and its relevance to the research question.

There were only 10 figures in the previous revised the manuscript, rather than 12. To address the issue of insufficient explanations, we have provided further supplementary clarifications for the corresponding figures in accordance with the suggestions, ensuring that the relationship between the content, variation trends, and research questions of each figure is explicitly explained. Specific details can be found in the red-marked text of the revised manuscript.

5. Regarding the sensitivity analysis comment, rather than doing the sensitivity analysis, the authors just revised the section name “Discussion about rural population change” and labeled it as a sensitivity analysis, but does this meet the expected standards? Please justify. A proper sensitivity analysis should include variation of model parameters across a plausible range with cross-splicing output evaluation. I believe the author should revise the name of this section, as this does not represent the as sensitivity analysis

In the previous revised manuscript, we only adjusted the title of the section "Discussion on rural population change," which stemmed from our understanding deviation for the concept of "sensitivity analysis". In fact, the core parameters of this simulation study are the birth rate and migration rate. In the latest revised manuscript, a section titled " The impact of births, deaths and migration rates on population change is updated. We adjusted the above-mentioned parameters within a reasonable range and systematically analyzed their effects on the population structure and change trends in the simulation results. From the perspective of the analytical purpose, this essentially aims to evaluate the responsiveness of the model output to changes in key input parameters, which is consistent with the core objectives of sensitivity analysis. Therefore, this section actually reflects the model's response characteristics to fluctuations in core parameters and possesses the essential attributes of sensitivity analysis. We consider it logically reasonable and well-founded to regard this as a sensitivity analysis of the model parameters.
6. The conclusion section is too long, please write a concise conclusion in 1-2 paragraphs.

We have refined the original conclusions with the core content while striving for concise language and a compact structure. To enhance the sense of organization and readability, we have maintained the form of point-by-point summarization, in which the first sentence of each point provides a more succinct generalization of the corresponding research conclusion.

**Reviewers' comments:**

The authors conducted a simulation and prediction of population using an agent based modelling on 3 factors: birth, death and migration. Overall, the manuscript is clearly written and presents a novel approach to the topic. However, several revisions are needed to improve the clarity and coherence of the presentation.

1. based on your response to reviewers, the validation part of Malthusian model is excluded in the latest manuscript, but in the abstract, it’s still showing ‘accuracy validated through Malthusian model’ (also see comment #6)

We would like to express our gratitude to the reviewers for carefully examining the details of the manuscript. In accordance with your suggestions, we have removed the expression " accuracy validated through Malthusian model " from the abstract of the first revised version. Additionally, we have made corresponding adjustments to the content of the abstract to ensure its consistency with the main body of the latest revised manuscript.

2. line 110-111, ‘(2) these models typically, these models concentrate on macro-level predictions…’ has a problem in sentence structure

In the second revised version of the manuscript, we have made adjustments to the grammatical structure of this sentence to enhance the accuracy and readability. The revised sentence is updated in line 70

3. line 120, the authors introduce intelligent agent approach rather abruptly. Although the authors mention a simulation approach in the previous paragraph, it’s better to add a clear transition to improve the flow.

We have carefully examined the structure of this paragraph and recognized that when introducing the agent-based modeling (ABM) approach, there is indeed a lack of necessary logical transition. To enhance the coherence between paragraphs, before introducing the ABM model, we have added a transitional sentence in line 73. This clearly points out that the deficiencies of traditional models have prompted researchers to turn to more flexible methods with the ability to represent individuals, thus naturally leading to the advantages of the agent-based model.
4. line 226-229, xi(t), bi, di can be typed using equation editor for clearer notations and properly aligned subscripts, like the authors do after equation 2

In accordance with your suggestions, we have re-entered the variables such as x_i_(t), b_i_, and d_i_ in lines 226–229 using a formula editor. This ensures that the subscripts and variable symbols are clear and standardized, and the typesetting is consistent. Meanwhile, we have also conducted a thorough check of the entire manuscript to ensure that the expressions of other similar mathematical symbols conform to the typesetting specifications.

5. the first paragraph of Population simulation section is a little confusing and contradictory. It seems like Xiache town is considered to be a relatively underdeveloped area (Guangdong for sure cannot be called the underdeveloped area), but it is also the provincial center and characterized by a high degree of specialization ad high incomes. Geographically, Heping county is located in the northeast part of Guangdong, and not really part of the pearl river delta. ‘the town serves as the provincial center’ - Xiache town is neither the capital of the Guangdong province nor able to serve as the center of province, given its limited resources.

We have rephrased the content of this paragraph, clearly pointing out that Xiache Town is located in Heping County, in the northeastern part of Guangdong Province. It is a typical township characterized by relatively backward economy and significant population outflow. We have deleted the ambiguous expression such as "provincial central town" and avoided the statement that equates the whole of Guangdong Province, so as to ensure that the content description is consistent with the actual geographical location and economic characteristics. In the article, Heping County is located in Heyuan City, and Heyuan City is an outer city of the Pearl River Delta. The intention of the expression in the article is not to claim that Xiache Town is the provincial capital or the provincial center, See line 193 for details.
6. tables 3-5 Malthus model results are NA, but in line 300-301, the authors mention ‘results were subsequently verified against the predictions generated by Malthus model’. If the results are not included and showed as NA only, the columns should be removed.

We would like to express our sincere gratitude for your valuable suggestions, esteemed expert. It is possible that the first revised version of the manuscript confused the reviewer. In the second-round revised version, all the relevant results and descriptions pertaining to the Malthusian model have been completely removed, and the related sentences have been updated accordingly.

7. tables 3-4, please add the unit for (Malthus model,) Leslile model, agent simulation, current population

We have made corresponding revisions to Table 3 and Table 4. The unit of the population in the tables has been uniformly marked as "persons". In addition, considering that the content related to the Malthusian model has been removed from the main text during the revision process, we have not retained the relevant information in the tables to avoid misunderstandings.

8. units: the authors sometimes use km, ha, %, ‰, as units. It would be better to keep the units consistent. Similarly, line 392, ‘the proportion of individuals aged 15–19 will continue to decrease, while the percentage of the population over 70 years will steadily increase.’ The sentence sounds a bit awkward. While proportion and percentage can be used interchangeably, they also refer to slightly different concepts.

We have unified the expressions of units throughout the entire text. The unit of the area of the relevant jurisdiction has been uniformly changed from km² to ha. For specific revisions, please refer to the section titled "Overview of the study area". In addition, concerning the problem in the expression of line 392, we have re-examined the logic and wording of that sentence and made revisions. We have used "proportion" as the unit for the population. For specific revisions, please refer to line 320 of the revised manuscript.

9. As the editor previously commented, the findings might not be generalizable given the study is based on two villages. The authors interpret the two villages as one with industrial advantages and the other without, both located in a relatively underdeveloped town. Using industrial (or economic) status alone might oversimplify the rural Chinese demographic diversity. Rural doesn’t necessarily imply underdeveloped, especially those close to major cities. Development level, accessibility, demographic profiles in rural China can be very different. Social, cultural, policy-related, educational and geographic factors can also contribute to rural population change. Nowadays some young people choose to migrate to rural areas because they want slower-paced lifestyle or lower living costs. Conversely, some people remain in their relatively poor remote villages because of strong family networks, ethnic identity or attachment to ancestral land. If a rural town's economy relies primarily on historical preservation, or national park tourism, will the town be classified as underdeveloped or industrially disadvantaged?

We acknowledge your concerns regarding the categorization of rural areas based on the development status of a specific industry. This approach may lead to oversimplification and may not fully capture the complex factors driving population changes in rural China. The sample selection of this study may not have reflected all kinds of rural types and the multi-dimensional causes influencing population changes.

In the study, two villages with typical characteristics but different industry levels were selected as case studies. These two villages respectively reflect different population migration scenarios caused by local industrial advantages and disadvantages, and such type differences are also widely prevalent in other similar rural areas across the country. We mainly classified two types of rural areas from the dimensions of industry and population development status, and conducted an in-depth exploration of the two different population migration situations. It is undeniable that various other factors can also lead to population changes in rural areas. Nevertheless, the focus of the analysis dimensions in this study lies in the impact of industry on the population, which has a certain degree of universality among rural areas nationwide.

In the revised manuscript, we have added a section titled "Limitations and prospects". Relevant content has been supplemented in the explanation of the research limitations, clearly pointing out that the number of village samples in the current study is limited. We also suggest that future research conduct model verification and application in a broader regional context to further enhance the adaptability and reliability of the simulation framework. For specific revisions, please refer to the relevant content after line 429.

Finally, regarding the question "If a rural town's economy relies primarily on historical preservation, or national park tourism, will the town be classified as underdeveloped or industrially disadvantaged?", my answer is negative. If the economy of a rural town relies on historical preservation or national park tourism, it is not necessarily classified as "underdeveloped" or "industrially disadvantaged". The key lies in whether its tourism industry has economies of scale, driving capabilities, stability, and the degree of integration with local social resources.

10. what are the study limitations?

Although agent-based modeling (ABM) provides an effective tool for depicting the relationship between the behaviors of microscopic individuals and macroscopic population dynamics, its application in simulating rural population changes still has certain limitations. Therefore, the limitations of the study have been supplemented in the revised manuscript, and the future research directions have been indicated. For specific revisions, please refer to the newly added section " Limitations and prospects", that is, the content after line 428.
